# Sorbitol‐Induced Synchronisation of *Babesia duncani* and Assessment of Linoleic Acid Effect on Parasite‐Derived Vesicles

**DOI:** 10.1111/pim.70034

**Published:** 2025-10-08

**Authors:** Simone Haak, Dong Chen, Ronald Soriano, Jonas Gunnarsson, Jose Thekkiniath

**Affiliations:** ^1^ Department of Biological Sciences Purdue University Fort Wayne Indiana USA; ^2^ Department of Civil and Mechanical Engineering Purdue University Fort Wayne Indiana USA; ^3^ Malvern Panalytical Westborough Massachusetts USA

**Keywords:** extracellular vesicles, human babesiosis, linoleic acid, synchronisation

## Abstract

Human babesiosis is an emerging infectious disease caused by a bloodborne single‐celled parasite belonging to the genus *Babesia.* Cases of human babesiosis are commonly reported in the United States, Western Europe and Asia*.* In the United States, the two major causative agents are *Babesia microti* and *Babesia duncani*. Transmitted to humans through tick bites, the parasite infects host red blood cells (RBCs). It induces flu‐like symptoms and has evolved mechanisms to manipulate the immune system, enabling its persistence. One key mechanism is the secretion of extracellular vesicles (EVs) which carry bioactive molecules, including proteins, lipids and genetic material that modulate pathogen–host interactions and disease development. The inhibition of the secretion of these vesicles may lead to disease control. One potential inhibitor of extracellular vesicle secretion is linoleic acid (LA), a polyunsaturated lipid that has demonstrated inhibitory properties in other parasites. To study the effects of development stage‐dependent stimuli on *B. duncani,* we employed a *B. duncani* in vitro continuous culture system and evaluated the use of sorbitol for synchronising parasite development. Microscopy techniques showed successful sorbitol‐induced synchronisation. Using nanoparticle tracking analysis and scanning electron microscopy, we assessed the effects of LA on parasite morphology and EV characteristics. Our studies indicate that exposure of *Babesia* parasites to LA did not cause significant observable changes in RBC morphology or reduce EV concentrations under the tested conditions.

**Abbreviations**
EV: extracellular vesicle
iRBC: infected red blood cell
LA: linoleic acid
NTA: nanoparticle tracking analysis
RBC: red blood cell
TH: th helper cells
uRBC: uninfected red blood cell

## 1 Introduction

Human babesiosis is an emerging infectious disease caused by the protozoan parasite *Babesia. Babesia* is a single‐celled bloodborne parasite within the phylum Apicomplexa [1]. This phylum contains some of the globe's most significant parasitic pathogens, such as *Plasmodium*, the causative agent of malaria. The major species that cause human babesiosis in the United States are *Babesia microti* and *Babesia duncani*. While many individuals infected with *B. duncani* remain asymptomatic, clinical manifestations range from mild symptoms, such as fever and muscle aches, to severe complications, including renal failure, splenic rupture and respiratory distress. In some cases, particularly among immunocompromised individuals, symptoms may evolve into organ failure and become fatal [1]. *Babesia* parasites are transmitted to humans via the bite of infected ticks during a blood meal. The stages of intraerythrocytic development for *B. duncani* consist of early rings (ER), mature rings (MR), filamentous forms (FF), young tetrads (YT) and mature tetrads (MT) [2].

During the FF stage of intraerythrocytic development, *B. duncani* secretes extracellular vesicles (EVs), which may play a crucial role in intracellular communication. Extracellular vesicles encompass a range of vesicle classes, including exosomes (50–100 nm), apoptotic bodies (1000–5000 nm) and microvesicles (MVs) (50–1000 nm) [3]. EVs are vital for cellular communication and have garnered significant attention for their potential as therapeutic tools and biomarkers [4]. EVs are implicated in modulating immune responses by promoting or suppressing immune activity. Studies indicate that EVs produced by pathogens carry a variety of biomolecules, such as genetic materials and metabolites, which interact with host immune cells to elicit a specific immune response [5].

Though the full content of EVs secreted by *B. duncani* is yet to be fully elucidated, a recent study has determined that EVs from *Babesia divergens*, which also causes infection in humans, contain microRNAs (miRNAs). Notably, certain miRNAs were upregulated in cultures with iRBCs as compared to uninfected RBCs (uRBCs), including miR‐4454, which has been linked to thrombopenia in *Plasmodium* infections [6, 7]. Other species of *Babesia* are known to secrete antigenic proteins by way of the secretory pathway and are directly exported to the RBC, negating the use of EVs or TOVs. One such protein is the variant erythrocyte surface antigen 1 (VESA1), which plays a role in antigenic variation and cytoadhesion and is expressed by *Babesia bovis*, which causes disease in cattle [8]. Another species that causes infection in humans, *B. microti*, has been found to secrete EVs containing antigenic protein BmGPI12 (also known as BmSA1), which has been used as a biomarker for active *B. microti* infection [9]. A previous study showed that B.microti EVs transport highly immunogenic antigens including BmGPI12 and BmIPA48 [10]. Although many proteins that may be transported by EVs by *Babesia* species are not yet known, they may have a pivotal role in mediating immune responses. Identifying inhibitors of EV secretion is vital to understanding the specific role of EVs in immune modulation.

While the body of knowledge surrounding EVs has been growing exponentially in recent years, some studies have explored potential inhibitors of EV secretion. One such inhibitor is linoleic acid (LA), which is a polyunsaturated fatty acid (PUFA) with two double bonds at the 9^th^ and 12^th^ carbons [11]. As an essential fatty acid, LA cannot be synthesised by the human body and must be obtained through diet [12]. Previous studies on mechanisms of PUFAs suggest that these unsaturated lipids influence the composition and fluidity of plasma membranes, particularly by inserting into the sn‐2‐acyl chain position of phospholipids, such as phosphatidylcholine (PC) and phosphatidylethanolamine (PE) [13]. The incorporation of unsaturated fatty acids disrupts membrane integrity, leading to the formation of holes, which increases the permeability of substances from the extracellular space [14].

Linoleic acid has been known to have suppressive effects on both *Leishmania donovani* membranous body secretion and *Plasmodium falciparum* growth in vitro [15, 16]. Clinical studies have linked LA‐rich diets to a shift from a Th‐2 immune response to a Th‐1 response, leading to reduced *L. donovani* macrophage infection and decreased EV secretion [17]. Notably, *B. duncani* shares both morphological and physiological traits with *P. falciparum*, suggesting that *B. duncani* may be susceptible to similar compounds like LA. Both *P. falciparum and B. duncani* infect the host's erythrocytes, which may be influenced by LA by impacting the membrane dynamics of the erythrocyte, which may make them similarly vulnerable to changes in lipid metabolism or signalling pathways induced by LA.

Here, we investigate the potential of LA to inhibit EV secretion by *B. duncani* in vitro. We also evaluate the use of sorbitol to synchronise *B. duncani*. The inhibitory potential of LA was further assessed using scanning electron microscopy (SEM) and nanoparticle analysis to examine potential morphological changes and EV concentrations.

## 2 Materials and Methods

### 2.1 *Babesiaduncani* WA‐1 Continuous in Vitro Propagation

Cryovials containing the WA‐1 (obtained from Dr. Choukri Ben Mamoun, Yale) were used to initiate propagation following a hypotonic washing. *Babesia duncani* was maintained at a haematocrit of 5% in human‐type O‐positive blood purchased from the American Red Cross. Parasites were cultured in a medium composed of Claycomb medium (Sigma 51800C) supplemented with 20% heat‐inactivated foetal bovine serum (FBS), hypoxanthine/thymidineT media supplement 2X (Sigma H0137), glutaminePlus Solution 1X (Atlanta Biologicals B90210), antibiotic/antimycotic 1X (Corning 30004CI) and 100 μg/ml gentamycin Reagent Solution (Gibco 15710‐064). Cultures were maintained in tissue culture plates in a growth chamber at 37°C under an atmosphere of 2% O_2_, 5% CO_2_ and 93% N_2_ as previously described [2].

### 2.2 Synchronisation of *B. duncani* Cultures Using Sorbitol

Sorbitol treatments were prepared at final concentrations of 0.001%, 0.005%, 0.01%, 1%, 1.5%, 2%, and 5% from an 18.2% (1 M) stock solution. Each concentration of sorbitol was added to triplicate wells containing *B. duncani* at 2% parasitaemia in 24‐well plates at 5% haematocrit. Synchronisation was assessed by sampling 30 μL from each well at designated time points, centrifuging at 200*g* for 12 s, and preparing thin blood smears, followed by Giemsa staining and microscopy. Smears for all treatment concentrations and control groups were prepared in triplicates at 0, 24 and 48 h post‐treatment. In an independent experiment, *B. duncani* cultures were exposed to 1% sorbitol for 10 min at identical culture conditions. The 1% concentration of sorbitol was determined to be optimal for synchronisation in preliminary experiments, while the 10‐min exposure time was selected based on studies in *P. falciparum* [18]. In this assay, the initial parasitaemia was subsequently set higher than in the primary synchronisation experiments, as *B. duncani* undergoes replication approximately every 22 h, and the shorter exposure period necessitated a higher initial parasitaemia to ensure that any effects of treatment with sorbitol would be readily observable.

### 2.3 Exposure of *B.duncani* in vitro cultures to LA

Linoleic acid was prepared at final concentrations of 0.1, 0.3, and 0.8 μM from a 99% stock solution (Thermo Scientific) and added to 1 mL of *B. duncani* cultured in vitro. Triplicate wells were prepared for each concentration and control, and thin blood smears were collected at 0, 24 and 48 h post‐treatment. Parasitaemia was quantified by light microscopy.

### 2.4 Isolation of EVs

Twelve wells, each containing 4 mL of *B. duncani* cultures at 10% parasitaemia, were taken from six‐well plates for EV isolation. Cultures were transferred to 15 mL Falcon tubes and centrifuged at 200*g* for 20 min at room temperature. The resulting supernatant was carefully collected, and was diluted with 5 mL of PBS, and centrifuged at 500*g* for 30 min at 4°C. The resulting supernatant was transferred to new tubes and was centrifuged at 16,000*g* for 45 min at 4°C. The supernatant was then aspirated and placed into 11.6 mL PA Ultracrimp tubes (Thermo Scientific Cat #: 03987). Samples were ultracentrifuged using a Sorvall WX Ultra 80 ultracentrifuge at 120,200*g* for 16 h at 4°C. The supernatant was discarded, and the isolated EV pellet was resuspended in PBS and maintained on ice until further use.

### 2.5 Treatment of Isolated EVs With LA

Isolated EVs were diluted in 15 mL of PBS, and 1 mL aliquots were distributed into 12 wells of a 24‐well plate. LA was diluted in PBS to final concentrations of 0.1, 0.3 and 0.8 μM, with control wells that did not receive LA. The plate was then incubated for 24 h at 37°C. Following incubation, the contents of each well were aspirated, transferred to eppendorf tubes, and centrifuged at 200*g* for 5 min to remove residual LA. The resulting pellet was resuspended in 1 mL of PBS and stored at −80°C until further analysis by NTA.

### 2.6 Nanoparticle Tracking Analysis

Nanoparticle tracking analysis (NTA) was employed to quantify EVs following direct treatment with LA using the NanoSight NS Pro system equipped with a 488 nm (blue) laser module. Prior to measurement, samples were further diluted 1:1000 in PBS to achieve proper volume and concentration for analysis. Flow mode was utilised to maximise particle detection, and PBS alone was measured as background. Data acquisition and analysis were performed using NS Xplorer software version 1.1, with five repeated measurements per sample, each consisting of 750 frames. This analysis was conducted in collaboration with Malvern Panalytical, a Spectris company, MA.

### 2.7 Scanning Electron Microscopy

*Babesiaduncani* was maintained in a continuous in vitro culture until parasitaemia reached approximately 10% before fixation. Following a 24 h incubation with LA, cultures were washed with Dulbecco's phosphate‐buffered saline (PBS) to remove residual culture medium and LA. Cultures were then centrifuged at 1500*g* for 7 min; the supernatant was discarded, and the RBCs were resuspended in 1 mL of PBS. The suspension was allowed to settle for 20 min. Primary fixation was performed by adding 0.5 mL of fixative containing 2% paraformaldehyde and 2.5% glutaraldehyde to samples for 30 min. Samples were placed onto glass coverslips and incubated for an additional 30 min. A secondary fixation with 2.5% glutaraldehyde in 0.1 M sodium cacodylate buffer (pH 7.4) was applied for 15 min, followed by washing with PBS. Post‐fixation was carried out with 2% osmium tetroxide in 0.1 M sodium cacodylate buffer (pH 7.4) for 15 min. Coverslips were washed once with PBS for 2 min, then washed twice with deionised water. Samples were dehydrated through a graded ethanol series (25%, 50%, 75%, 90%, 95% and 100%) for 5 min each, then dried to the critical point. Finally, specimens were sputter‐coated with 5 nm gold under vacuum and examined using a Hitachi S‐3400 N scanning electron microscope operating at 10.0 kV [19, 20].

## 3 Results

### 3.1 Synchronisation of *B. duncani* Cultures Using Sorbitol

**FIGURE 1:**
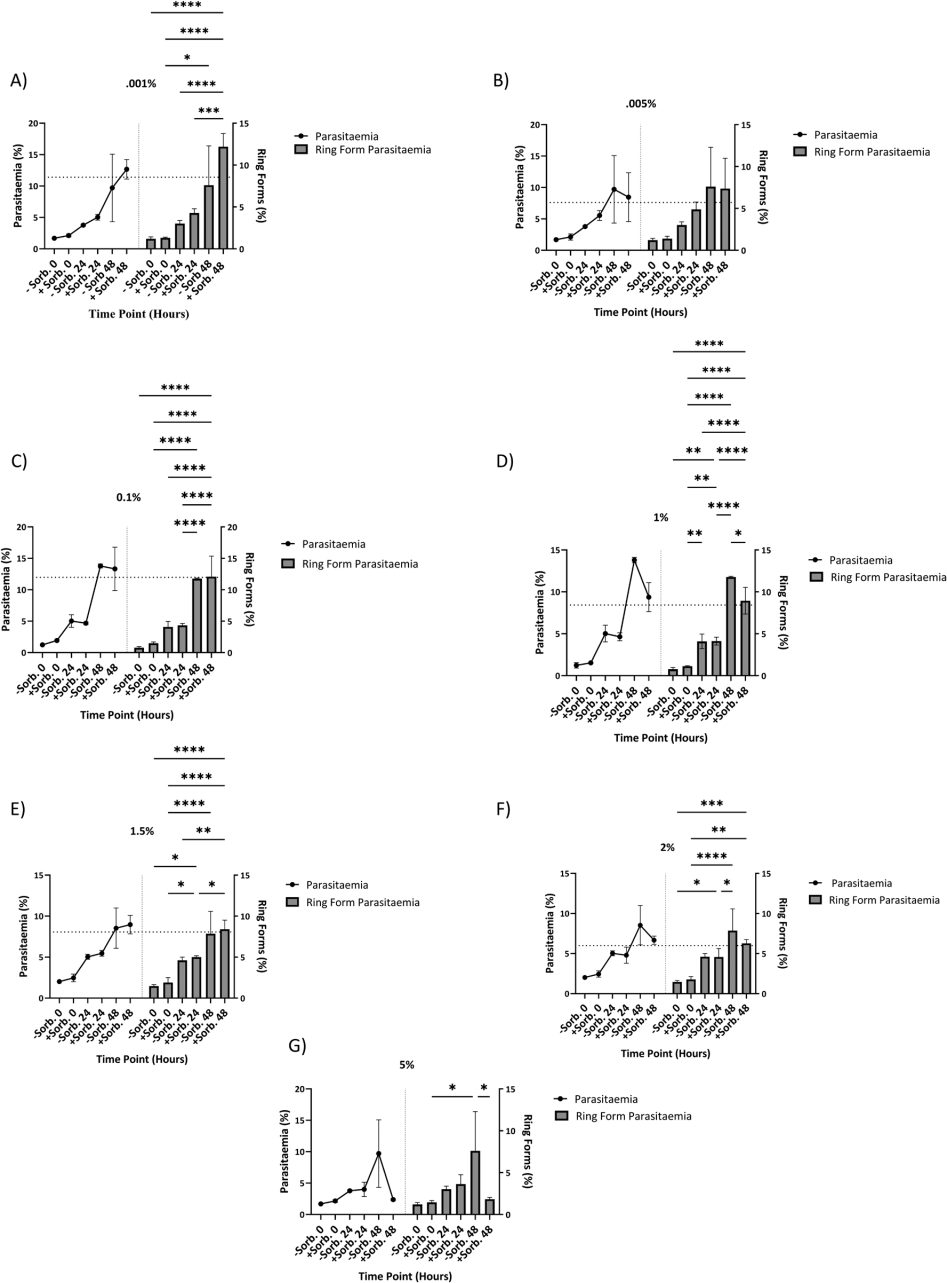
Synchronisation of *B. duncani* in vitro following treatment with various concentrations of sorbitol, 0.001% (A), 0.005% (B), 0.1% (C), 1% (D), 1.5% (E), 2% (F) and 5% (G). Total parasitaemia versus ring form parasitaemia at 0, 24 and 48 h for both treatment and control groups is shown. Results were analysed by two‐way ANOVA with repeated measures using GraphPad Prism 9.0 with (*p* < 0.05) (*0.0260) (***0.0010) (**** < 0.0001) (ns = not significant) (*0.0124) (**0.0018, 0.0074, 0.0010) (**** < 0.0001), (*0.0175, 0.0499, 0.0157) (**0.0024) (*0.0342, 0.0237) (**0.0012) (***0.0005) (**** < 0.0001) (*0.0149, 0.0265). A threshold of 90% of the treatment group total parasitaemia at 48 h was added to each graph as represented by a dashed line. Parasitaemia is represented by grey dots vs. grey bars for ring form parasitaemia, with error bars with standard deviation.

The structure of LA is shown (Figure S1). To determine the optimal concentration of sorbitol for synchronising *Babesia duncani*, a range of concentrations (0.001%, 0.005%, 0.01%, 1%, 1.5%, 2% and 5%) were tested. Total parasitaemia is represented by connected black dots, and the ring‐stage parasitaemia, excluding other life stages (FF, YT and MT), is indicated by grey bars across all concentrations and time points. A 90% ring‐stage synchronisation threshold was set to evaluate effectiveness. The concentration of sorbitol that achieved both the highest overall parasitaemia, alongside > 90% ring‐stage parasitaemia, was determined to be 1% sorbitol at 28 h post‐treatment (Figure 1). At 0.001% sorbitol, total parasitaemia increased over 48 h in both treated and untreated groups, with a corresponding increase in ring‐stage parasitaemia correlating with total parasitaemia (Figure 1A). Similarly, at 0.0005% sorbitol, total parasitaemia approximately doubled as expected, peaking at 10% total parasitaemia at 48 h in untreated controls. Despite the increase, ring‐stage parasite parasitaemia remained relatively consistent between the treatment and control groups (Figure 1B).

When the cultures were exposed to 0.1% sorbitol, the total parasitaemia exhibited a steady increase from 0 to 24 h, followed by an unexpected threefold increase between 24 and 48 h; this trend was consistent between the treatment and control groups. Ring‐stage parasitaemia followed a similar pattern, with no significant difference observed between the treatment and control groups (Figure 1C). At 1% sorbitol, total parasitaemia increased steadily in both treated and control groups; however, at 48 h, treated cultures exhibited lower total parasitaemia but achieved > 90% ring‐stage synchronisation. This suggests possible osmotic stress or pathway interruption induced by sorbitol, leading to effective synchronisation (Figure 1D).

At 1.5% sorbitol, the total parasitaemia was increased twofold between 0 and 24 h and 24 and 48 h as anticipated; however, the ring‐form parasitaemia remained consistent between treatment and control groups at all time points, indicating that 1.5% sorbitol is not the optimal concentration for synchronisation (Figure 1E). At 2% sorbitol, treated groups exhibited lower total parasitaemia compared to controls at both 24 and 48 h following treatment. The ring‐stage parasitaemia reflected this trend with a lower percentage of parasites synchronised to the ring stage.

Finally (Figure 1F), at 5% sorbitol, total parasitaemia was comparable between treated and control groups until 48 h post‐treatment, where parasitaemia peaked at 8% in untreated wells as compared to 6% in treated wells, with a similar decrease in ring form parasitaemia (Figure 1G).

**FIGURE 2:**
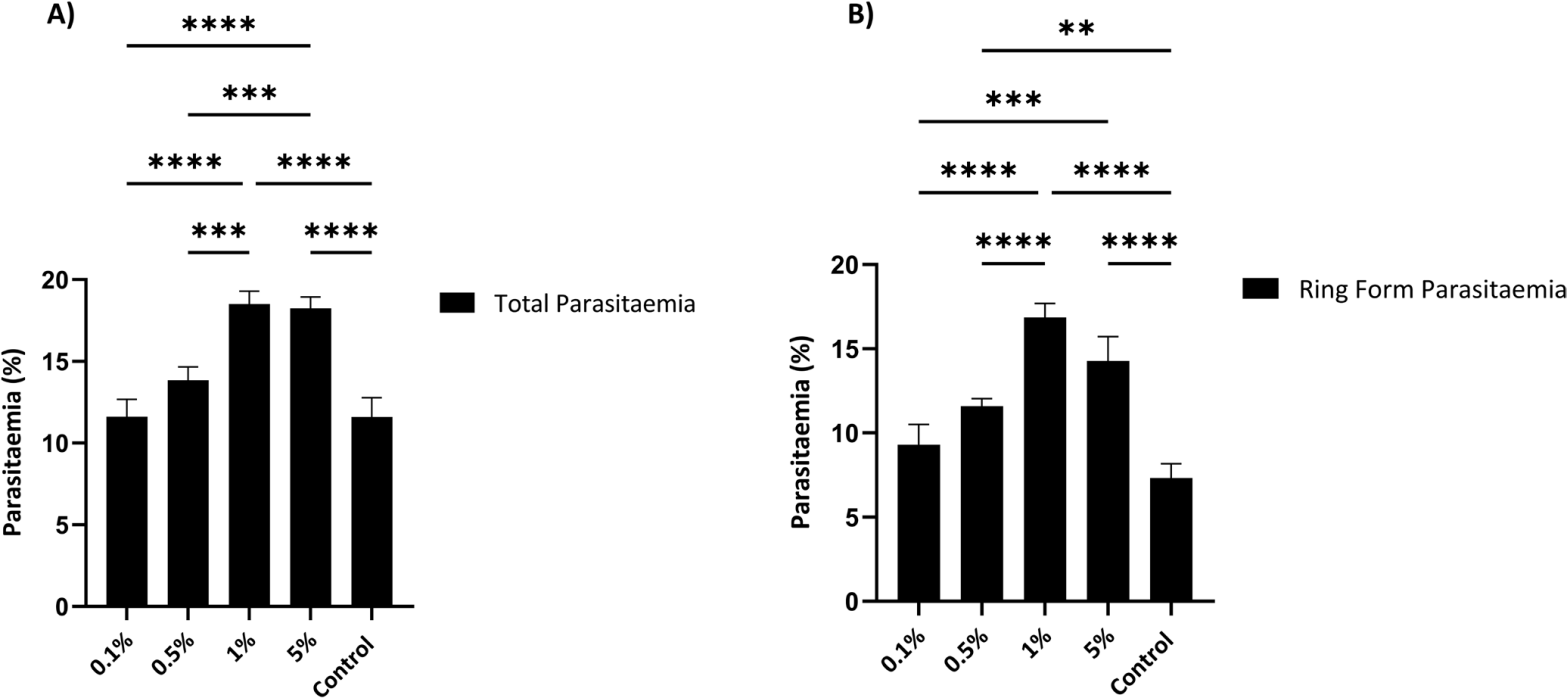
Synchronisation of *Babesia duncani* in vitro following 10 min of incubation at sorbitol concentrations of 0.1%, 0.5%, 1% and 5%, compared to the control, which did not receive sorbitol. The total parasitaemia (A), and the ring form parasitaemia (B) determined by blood smears, are represented by black bars and presented as a percentage. Results were analysed by two‐way ANOVA with repeated measures using GraphPad Prism 0.9 with (*p* < 0.05) (**** < 0.0001) (***0.0007) (***0.0003) (***0.0002) (**0.0010).

To assess the effects of sorbitol at a shorter incubation period, 0.1%, 0.5%, 1% and 5% sorbitol were incubated with *B. duncani* for 10 min (Figure 2). Total parasitaemia after treatment was approximately 12% for concentrations 0.1% and 0.5%. Groups that received 1% and 5% sorbitol displayed a total parasitaemia of approximately 18%. The total parasitaemia for the untreated control was approximately 12%, comparable to that of the lowest sorbitol concentration (Figure 2A). The corresponding ring‐stage parasitaemia increased progressively with sorbitol, peaking at 1% and decreasing at 5%. The ring‐stage parasitaemia of the control group was lower than that of the lowest concentration of sorbitol, suggesting a minor but non‐significant effect of the lowest concentration.

### 3.2 Exposure of *B. duncani* Cultures to Linoleic Acid

**FIGURE 3:**
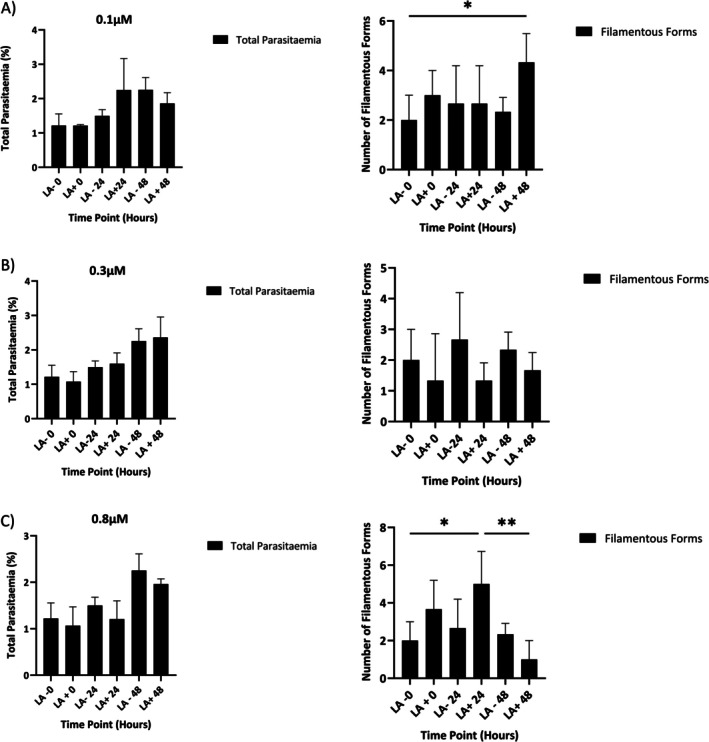
Effect of LA on *B. duncani* total parasitaemia vs. the number of filamentous forms (FF) at 0, 24 and 48 h with (LA+) and without (LA−) at different concentrations 0.1 μM (A), 0.3 μM (B), 0.8 μM (C). Analysed by two‐way ANOVA on GraphPad Prism 9.0 (*p* < 0.05) (*0.0392) (**0.0024) (*0.0214).

The effects of LA on total parasitaemia and the development of FF in *B. duncani* were assessed at a range of concentrations of 0.1, 0.3 and 0.8 μM, with measurements taken at 0, 24 and 48 h for both control and treatment groups (Figure 3). At 0.1 μM, the total parasitaemia was approximately 1.5% for both treatment and control groups at hour 0. By 24 h, the treatment group exhibited higher total parasitaemia, as compared to the total parasitaemia of the control group. The total parasitaemia for the control group was similar to the total parasitaemia for the treated group at 24 h, suggesting delayed growth. The number of FF remained relatively stable between the control and treatment groups over 48 h, except for an increase at 48 h for the treatment group. Our results suggest that 0.1 μM is suboptimal for limiting FF development, which is associated with the successful production and secretion of EVs.

At a total concentration of 0.3 μM, the total parasitaemia increased steadily and similarly in both treatment and control groups, suggesting that LA at this concentration does not significantly affect parasite growth. Despite the increase in parasitaemia, the number of observed FF remained consistent between groups, indicating that 0.3 μM LA may have a mild effect on the progression of parasites to the FF stage, wherein EVs are produced (Figure 3B).

At 0.8 μM LA, total parasitaemia was consistently higher in controls than in treated groups at all time points, indicating a modest inhibitory effect of LA at this concentration. Similarly, the number of FF was reduced in each of the treatment groups as compared to the control groups across all time points. This reduction may reflect direct effects of LA on FF or secondary effects related to decreased parasitaemia (Figure 3C). Collectively, our results suggest that 0.8 μM LA is the optimal concentration for an observable decrease in parasites that reach the FF life stage, thereby potentially decreasing EV secretion.

### 3.3 Treatment of Isolated EVs With LA

**FIGURE 4:**
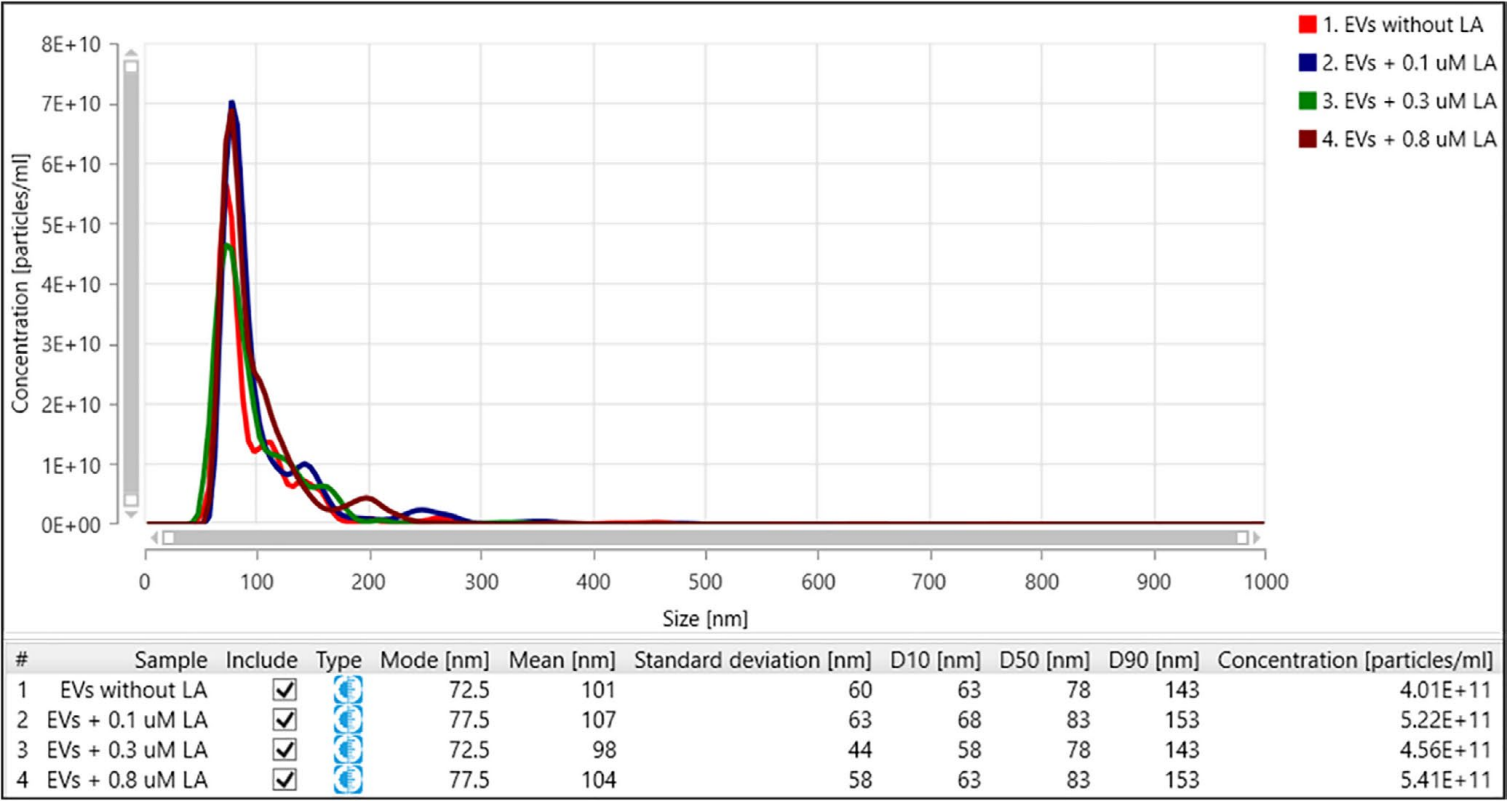
Nanoparticle tracking analysis of extracellular vesicles isolated from *Babesia duncani* culture with and without linoleic acid treatment. Table shows mean size, mode size and particle concentration for EVs under four conditions: control (EVs and PBS only), 0.1, 0.3 and 0.8 μM LA. Data represent mean ± SD from five repeated measurements per sample. All samples displayed a predominant exosome population. Measurements were taken by NanoSight NS Pro.

To evaluate whether LA significantly impacts EV concentrations, isolated EVs from *B. duncani* in vitro were exposed to a range of concentrations of LA followed by NTA. Each sample was measured in five replicates, and particle size distribution was assessed to confirm the presence of exoso secreted by *B. duncani*. The mode size of EVs ranged from 73 to 78 nm, with a mean size of 98–107 nm. This was consistent with the presence of exosomes rather than larger MVs or apoptotic bodies (Figure 4).

There were four samples tested containing EVs treated with 0.1, 0.3 and 0.8 μM LA, and a control containing only EVs with PBS diluent. The concentration of EVs following direct contact with LA was similar between all four samples, with the highest concentration observed in the 0.8 μM LA sample at 5.4 x 10^11^ particles/mL, indicating that this concentration is not effective at inhibiting EVs. The lowest concentration of EVs was measured in the untreated control sample. The 0.1 and 0.3 μM LA‐treated samples contained 5.2 x 10^11^ and 4.6 x 10^11^ particles/mL, respectively. These findings suggest that LA does not effectively reduce EV concentrations through direct interaction (Figure 4).

### 3.4 Scanning Electron Microscopy Images of *B. duncani* Following Treatment With LA

**FIGURE 5:**
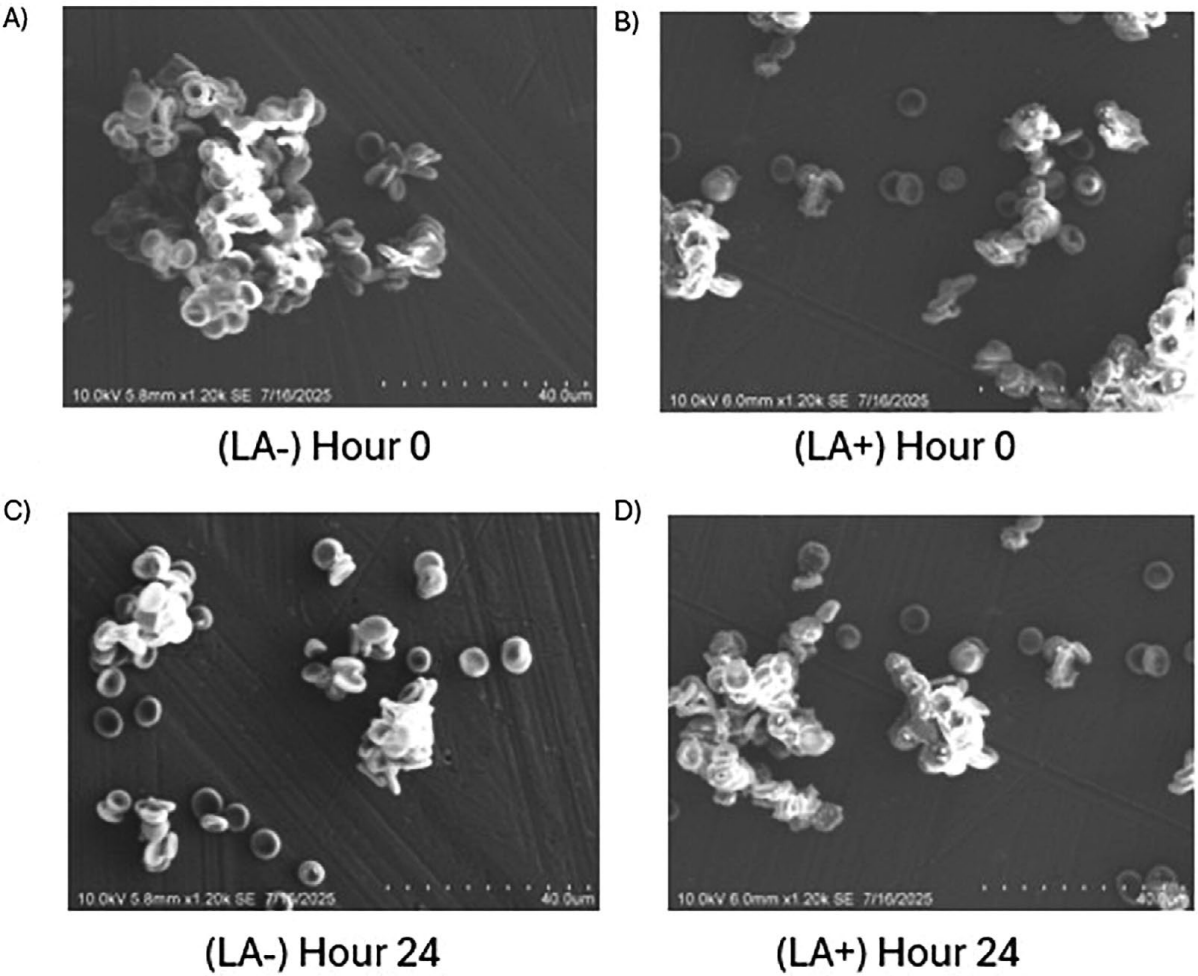
Scanning electron microscopy (SEM) images of *B. duncani* in vitro. (A) without treatment at hour 0. (B) at 0 h post treatment with LA. (C) without treatment, at 24 hours. (D) 24 h following treatment with LA at 10 kV.

Scanning electron microscopy was employed to examine the life stages of *B. duncani* and to assess potential morphological changes in red blood cells following treatment with LA. The results demonstrated that the direct exposure of LA to *B. duncani* in vitro did not induce any observable alterations in the surface morphology of infected or uRBCs. Furthermore, distinct intraerythrocytic development stages were additionally not observed under these conditions (Figure 5A–D).

### 3.5 Light Microscopy Blood Smears

**FIGURE 6:**
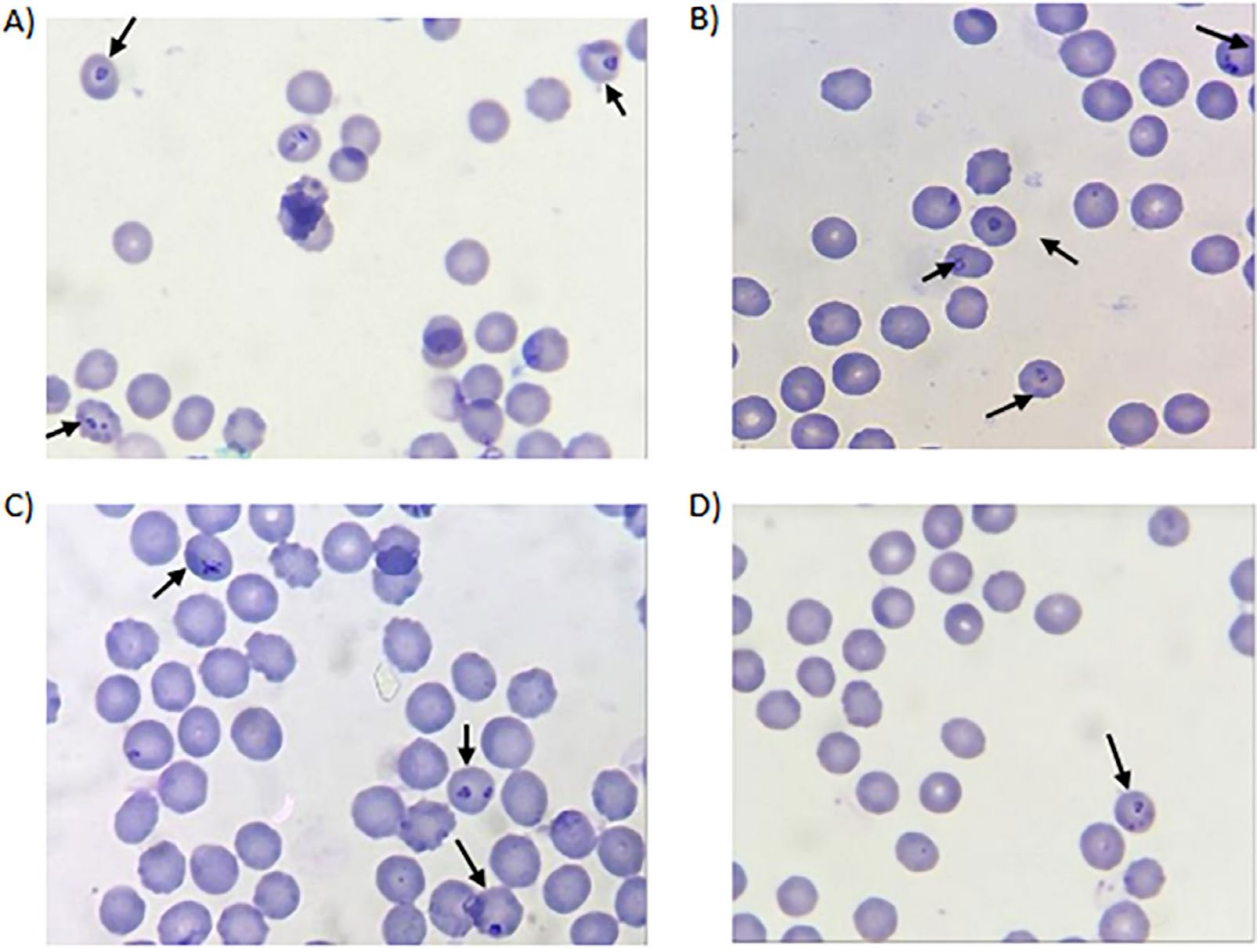
(A) *Babesia duncani* in vitro with 1% sorbitol at 48 h of incubation. The synchronised intracellular ring stage parasites are indicated by black arrows. (B) *B. duncani* in vitro without sorbitol at 48 h of incubation with filamentous form and ring stage parasites. (C) *Babesia duncani* in vitro without 0.8 μM LA at 48 h, with FF and ring form parasites. (D) *Babesia duncani* in vitro at 48 h with 0.8 μM LA with ring form parasites.

Blood smears of *B. duncani* were prepared to evaluate synchronisation and parasite development. Cultures treated with 1% sorbitol at 48 h exhibited predominantly synchronised ring‐form parasites. In contrast, untreated cultures displayed a mixture of ring‐stage and FF parasites, reflecting an unsynchronised population. Similarly, cultures without 0.8 μM LA contained both FF and ring‐form parasites, whereas treatment with 0.8 μM LA inhibited FF development, which corresponded with a decrease in overall parasitaemia (Figure 6A–D).

## 4 Discussion

Extracellular are produced during the filamentous form stage of intraerythrocytic development in *B. duncani*. These vesicles are increasingly recognised as key mediators in parasite–host interactions, contributing to the pathogenesis of human babesiosis. Therefore, regulating EV production holds significant potential for understanding disease mechanisms and identifying therapeutic targets. Synchronising *B. duncani* parasites may provide a reliable approach to study the specific effects of LA on both FF development and EV secretion, by promoting uniform parasite development in vitro and thereby reducing experimental variability. Sorbitol‐induced synchronisation is a well‐established method in *Plasmodium* research and has been employed to enrich ring‐stage parasites in *P*.*falciparum*, *B*.*divergens*, and *B*. *bovis* [18, 21, 22]. Sorbitol, a sugar alcohol impermeable to intact erythrocyte membranes, selectively enters the iRBCs via new permeability pathways (NPPs) that develop predominantly during the trophozoite stage of *Plasmodium falciparum* [23, 24]. These NPPs facilitate nutrient uptake and waste elimination in *Plasmodium* spp.,To but render infected RBCs susceptible to osmotic lysis when exposed to sorbitol [25]. This effectively synchronises parasites by selectively lysing more mature stages and sparing ring forms [26]. However, a previous study showed that despite similarities between *Plasmodium* spp. and *Babesia* spp., *Babesia* does not employ NPPs to uptake nutrients and promote survival and instead alters the permeability of host membranes through non‐channel‐based mechanisms. While the exact molecular details of these methods are largely uncharacterised, it has been suggested that the modification or disruption of lipid populations, different parasite protein interactions with the erythrocyte membrane, or alterations to the RBC cytoskeleton may play significant roles in this process [21].

To apply sorbitol‐induced synchronisation to *B. duncani*, we tested a series of concentrations in vitro and assessed synchronisation efficiency by quantifying ring‐stage parasitaemia over time. Among tested concentrations, 1% sorbitol at 48 h post‐treatment was optimal, yielding nearly 10% total parasitaemia with over 90% ring‐stage synchronisation, consistent with parasite replication doubling approximately every 22 h. This suggests that the osmotic properties of sorbitol effectively select for ring‐stage parasites in *B. duncani*, similar to mechanisms described for *Plasmodium falciparum*. Additionally, shorter sorbitol incubations at selected concentrations, including 1%, maintained effective synchronisation, consistent with the previous study in *P. falciparum* supporting shorter exposure to sorbitol for effective intraerythrocytic synchronisation [18]. While comparing the percentages of ring‐stage parasites from treatments with 1% sorbitol at 48 h of incubation to 10 min, there is an increase in the ring‐stage parasitaemia after 10 min of incubation. This indicates that shorter periods of incubation at 1% sorbitol are most effective for the synchronisation of *B. duncani* parasites.

Investigation of LA's impact on parasite development focused on its influence on FF formation, a proxy for EV production. Across tested concentrations, statistical analysis revealed no significant differences in total parasitaemia between treatment and control groups, indicating that LA does not substantially impair overall parasite growth. However, a reduction in filamentous forms was observed at 0.8 μM LA after 48 h, with the treated cultures showing approximately one‐third the number of FF compared to controls, which suggests the potential of LA to inhibit EV‐associated developmental stages. The underlying mechanism may involve increased membrane fluidity from the incorporation of the unsaturated fatty acid into the RBC membrane and the potential disruption of pathways critical for FF formation or EV secretion. Scanning electron microscopy corroborated these findings, showing no major morphological alterations in the surface of infected and uninfected RBCs following treatment with LA, though cells that received LA experienced increased membrane blebbing. This could be due to the biophysical effects of LA on the plasma membrane of RBCs, which may influence EV secretion without overt structural damage.

To determine whether LA has a direct effect on EVs aside from biochemical pathways of the RBC membrane, we isolated EVs from *B. duncani* in vitro and incubated them with a range of concentrations of LA and quantified the resulting EV concentrations by NTA. This analysis further demonstrated that the direct exposure of EVs to LA did not reduce EV concentrations. This may be due to differences in the composition of EVs and RBC membrane and may indicate that modulatory effects occur at the level of parasite development rather than affecting EVs extracellularly. To better understand the modulatory effect of LA on EVs, optimisation with additional exposure time points and concentrations of LA may be needed.

Overall our results showed that 1% sorbitol efficiently synchronised *B. duncani* to the ring stage at 10 min rather than 48 h. This was observed as a comparison between the percentage of ring‐stage parasites relative to total parasitaemia at both time points. We also investigated the potential of LA to inhibit EV secretion by *B. duncani* in vitro and concluded that while LA does not directly reduce EV concentrations, it may exert inhibitory effects on the development of filamentous forms, implicating a possible novel role in modulating parasite maturation and EV secretion. These insights may lay the groundwork for future studies to explore the therapeutic potential of manipulating parasite lipid environments to mitigate babesiosis pathology.

## Conflicts of Interest

The authors declare no conflicts of interest.

## Peer Review

The peer review history for this article is available at https://www.webofscience.com/api/gateway/wos/peer‐review/10.1111/pim.70034.

## Supporting information


**Figure S1:** pim70034‐sup‐0001‐FigureS1.docx.

## Data Availability

The data that support the findings of this study are available on request from the corresponding author. The data are not publicly available due to privacy or ethical restrictions.
