# Supplementary figures and images for: Sorbitol‐Induced Synchronisation of Babesia duncani and Assessment of Linoleic Acid Effect on Parasite‐Derived Vesicles
Source: Parasite Immunol. 2025 Oct 8;47(10):e70034. doi: 10.1111/pim.70034 (PMC12505203; doi:10.1111/pim.70034)

Supplementary file

Figure S1


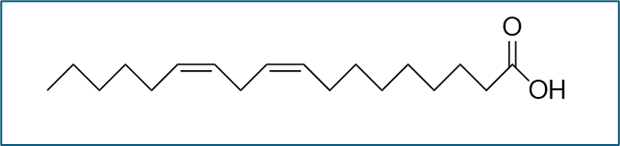

Supplement: Supplementary file 1 — Figure S1: pim70034‐sup‐0001‐FigureS1.docx. [file PIM-47-e70034-s001.docx]
